# Fertility patients’ use and perceptions of online fertility educational material

**DOI:** 10.1186/s40738-020-00083-2

**Published:** 2020-07-18

**Authors:** Claire Ann Jones, Chaula Mehta, Rhonda Zwingerman, Kimberly E. Liu

**Affiliations:** 1 Mount Sinai Fertility, Sinai Health System, 250 Dundas St. West, Suite 700, Toronto, Ontario M5T 2Z5 Canada; 2 Department of Obstetrics and Gynaecology, University of Toronto, 123 Edward St., Suite 1200, Toronto, Ontario M5G 1E2 Canada

**Keywords:** Counselling, Fertility, Infertility, Patient education

## Abstract

**Background:**

Online educational information is highly sought out by patients with infertility. This study aims to assess patient-reported usage and helpfulness of fertility educational material on a clinic website and social media accounts.

**Methods:**

Educational material was created on common fertility topics in text and video format and posted on the clinic website and social media accounts. At the first consultation for infertility, patients were provided with a postcard directing them to material online. At the first follow-up appointment, patients were invited to fill out a survey assessing whether patients viewed the online educational material and if they found the information helpful.

**Results:**

98.4% (251/255) of patients completed the survey, of which 42.6% (106/249) looked at the online material. Of those who viewed the online information, 99.1% (115/116) found the information helpful or somewhat helpful and 67.6% (73/108) found reading the material online better prepared them for making fertility decisions at their doctor’s appointment

**Conclusion:**

Patients found online fertility information on the clinic website and social media accounts useful for making fertility treatment decisions. Providing online educational material has the potential to improve patient care by empowering patients with the knowledge to make more informed treatment decisions, and improving the quality of the time spent with the physician.

## Background

In the current age of easy access to the internet and widespread use of social media, patients often search online for information on medical conditions while concurrently seeking the advice of a physician [1, 2]. Online information can be biased in favour of a discussion of benefits over risks, lack of evidence-based research to back up recommendations, and fail to disclose conflicts of interests, especially when most information has been created by private companies rather than physicians [3–6]. Even among fertility clinics’ websites, the information provided can be biased and incomplete with little discussion of safety and risks, particularly for newer technologies such as pre-implantation genetic testing [7]. Furthermore, most online medical information is written at an educational level that is too high for the average patient to understand [8–10].

While many young people are misinformed about age-related fertility decline, providing this information has the potential to affect decisions to delay child-bearing and therefore avoid unintended childlessness [11–15]. Only a small proportion of fertility patients, however, are aware of the dramatic impact of age on fertility, suggesting that current information provision is insufficient [11–13]. While online information has its limitations, it is still highly sought out by fertility patients who find that the social connection online can help to make them feel less isolated and abnormal [16]. It is imperative that fertility doctors have reliable online resources to which they can direct their patients. One way to achieve this is for physicians to create accurate, high quality online educational material, which has been successful in a number of different areas of medicine [17–22]. We designed online patient information for our fertility clinic website that was intended to be both comprehensive and evidence-based.

The purpose of the study was to assess patient-reported usage and helpfulness of fertility educational material that was created by our university-affiliated hospital-based fertility clinic doctors and posted on our clinic website and social media accounts.

## Methods

Online educational materials (video and text-based) were created on the following common fertility topics: (1) causes and treatment of infertility, (2) polycystic ovarian syndrome, (3) fertility medications, (4) assisted reproductive technologies, and (5) optimizing natural fertility. The information was created by two study investigators based on a review of the medical literature and extensive experience counselling patients on these topics. The information was reviewed for content and readability which was assessed to vary between a grade 6 and a grade 10 level, and was edited by all physicians at the fertility clinic and by a sample of patients and administrative staff. The materials were provided in both text and video format on the fertility clinic website, Facebook and Twitter sites (Additional file 1).

A patient survey was created by two study investigators (CJ and KL) after reviewing the literature for similar survey designs [23]. The goal of the survey was to assess the usefulness of the online educational material. The survey was circulated among physicians at the fertility clinic for feedback and to a small number of non-medical staff to comment on readability. The survey was presented to physicians, nurses and administrative staff at the fertility clinic for additional feedback before the final version was set.

People attending an initial infertility consultation were given an information postcard about the online educational material. The card included the website addresses for the clinic, Twitter and Facebook pages, and QR codes that linked to the videos. Whenever an information card was given to a patient, a survey was placed in the patients chart with a cover page reminding the clinic secretary to invite the patient to participate in the study and fill out the survey at the subsequent appointment.

At the subsequent appointment, clinic secretaries invited patients to fill out the voluntary, anonymous paper-based survey (Additional file 2). When a patient was seen together with a partner, only one of them completed the survey. A sample size of 250 patients was chosen based on previous studies with a similar design.

The survey included questions about baseline demographics, access to the educational material, and its usefulness. Descriptive statistics were used to summarize demographic data (Table 1). Demographic data was compared between patients who answered the question about whether they had viewed or had not viewed the online educational material for mean age, gender, ethnicity and education using t-tests for continuous variables and chi-squared tests for categorical variables. Chi square tests were used to compare differences in the proportions of patients who had preferences for video-based content and support for posting of more online information between those who had accessed information online and those who had not.

**Table 1:** Baseline demographics of survey respondents

	**Total** **N (%)**	**Accessed online educational material** **N (%)**	**Did not access online educational material N (%)**	***P*-value**
**Mean Age (SD)**	34.5 (4.6)	34.3 (4.5)	34.6 (4.8)	0.5600
**Gender**				0.0856
Male	14/251 (5.6%)	9/106 (8.49%)	5/145 (3.45%)	
Female	237/251 (94.4%)	97/106 (91.51%)	140/145 (96.55%)	
**Ethnic background**				0.1448
Asian	58/251 (23.1%)	21/106 (19.81%)	37/145 (25.52%)	
Caucasian	143/251 (57.0%)	58/106 (54.72%)	85/145 (58.62%)	
Other	50/251 (19.9%)	27/106 (25.47%)	23/145 (15.86%)	
**Highest Level of Education**				0.2789
High School	13/250 (5.2%)	4/106 (3.77%)	9/144 (6.25%)	
College	54/250 (21.6%)	18/106 (16.98%)	36/144 (25.00%)	
Undergraduate Degree	79/250 (31.6%)	34/106 (32.08%)	45/144 (31.25%)	
Graduate Degree	61/250 (24.4%)	32/106 (30.19%)	29/144 (20.14%)	
Professional Degree	43/250 (17.2%)	18/106 (16.98%)	25/144 (17.36%)	

Statistical analysis was performed using Statistical Analysis System, Version 9.4 (SAS Institute, Cary, North Carolina, USA). At the end of the survey, respondents were invited to suggest topics for additional online educational material. The responses were coded for qualitative analysis and grouped by common themes.

This study was approved by the Mount Sinai Hospital Research Ethics Board (REB # 130264-E).

## Results

**Fig. 1:**
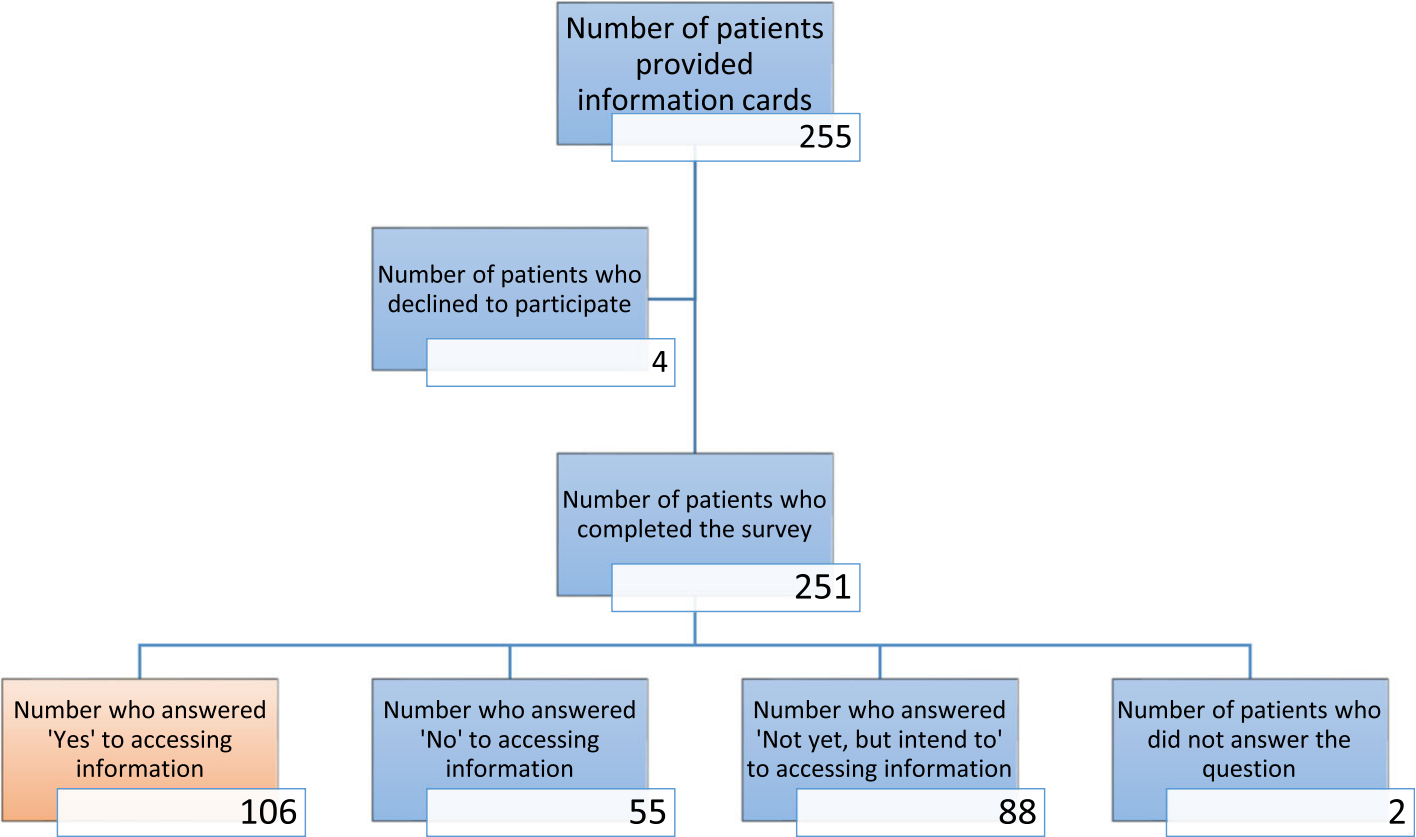
Flow diagram of study participants

Between January 2014 and May 2017, 255 patients were provided information cards at their initial appointment and invited to participate in the study at the subsequent clinic visit. Four patients declined to participate leaving 251 who completed the survey (Fig. 1).

One hundred and six respondents (42.6%) stated that they had accessed the educational material. The remainder of participants who answered “No”, “Not yet, but intend to” and the 2 participants who did not answer the question were grouped together as not having accessed online information for the purposes of statistical analysis (Fig. 1). Forty-seven out of the 251 (18.9%) patients did not recall receiving an information card. Of the respondents who did not view the materials, the reasons they gave were: not enough time (74.1%), already well informed (10.3%), went to other sites (7.8%), not interested (1.7%), and forgot (6.0%). Baseline demographics were not significantly different between those people who had stated that they had accessed online information to those who had not (Table 1).

Table 2 lists the survey questions along with their responses. The majority (95.8%) of respondents who viewed the online information stated that they accessed it through the clinic website, 1.7% through Facebook, and 2.5% on Twitter. Ninety-nine percent of patients stated that they found the information helpful or somewhat helpful and 67.6% stated that reading the material online better prepared them for making fertility decisions at their doctor’s appointment (Table 2).

**Table 2:** Responses to survey questions about online educational material

	**n (%)**
**Questions for those who viewed online material (*n*** **= 106)**	
Where did you go to find the online material?	
• Clinic website	114/119 (95.8)
• Twitter account	3/119 (2.5)
• Facebook account	2/119 (1.7)
Did you find the information helpful?	
• Very helpful	85/116 (73.3)
• Somewhat helpful	30/116 (25.9)
• Not helpful	1/116 (0.9)
Was the length of the information appropriate?	
• Too long	7/102 (6.9)
• Perfect length	93/102 (91.2)
• Not long enough	2/102 (2.0)
Was the information easy to read?	
• Very easy	31/107 (29.0)
• Easy	73/107 (68.2)
• Somewhat confusing	3/107 (2.8)
• Very confusing	0/107 (0)
Which educational material did you look at?	
• Text	23/109 (21.1)
• Videos	56/109 (51.4)
• Text and Videos	30/109 (27.5)
Did reading material online before this appointment better prepare you for making fertility decisions today?	
• Yes	73/108 (67.6)
• No	19/108 (17.6)
• Unsure	16/108 (14.8)
**Questions for all survey respondents (*n*** **= 251)**	
Which format do you prefer for online educational material?	
• Text	43/186 (23.1)
• Videos	122/186 (65.6)
• Text and videos	21/186 (11.3)
Would you like us to post more educational information online?	
• Yes	127/177 (71.8)
• No	7/177 (4.0)
• Unsure	43/177 (24.3)
Provide suggestions of topics that you would like to see online	
• Other general fertility topics	6/28 (21.4)
• Donor gametes and adoption	2/28 (7.1)
• Emotional support and counseling	2/28 (7.1)
• How-to videos and interactive process-related videos	3/28 (10.7)
• Risks of fertility treatment and pregnancy	2/28 (7.1)
• Patient stories	3/28 (10.7)
• Not sure	3/28 (10.7)
• Other	7/28 (25.0)

Participants preferred video-based content over text based content (65.6% vs. 11.3%). There were no differences in the preference for video-based content between those who had accessed the information online compared to those who had not (65.6% vs. 65.6%, *p* = 0.8229). Seventy-two percent of participants stated that they would like to see more information posted online (Table 2). Respondents who viewed the online material were more likely to support the posting of more online information (79.4% vs. 62.5%, *p* = 0.009).

Qualitative analysis was performed for the 28 participants who provided written comments suggesting future topics for online educational material. These comments were coded and grouped into the following themes: detailed information on infertility, fertility testing and fertility treatment (28.6%), improving natural fertility and use of alternative therapies (17.9%), donor gametes and transgender fertility care (14.3%), step-by-step approach (14.3%), emotional support and counseling (10.7%), patient stories (10.7%), not sure (10.7%) and other (14.3%).

More detailed information about causes of infertility, fertility testing and interpretation and fertility treatment including risks and the chance of conception with various treatment options by age was suggested by 8 participants. Specific suggestions included detailed information on “ovarian failure”, “AMH”, “age statistics” and “genetics”. One patient recommended a “glossary of medical terms”.

Five participants were interested in learning more about improving natural fertility, including the use of “alternative therapies” and “natural fertility aids”. Patients wanted to know what additional things they could do to support their fertility treatment to “improve chances getting a healthy baby”.

Information on donor gametes was recommended by 3 participants and information on transgender fertility care was recommended by 1 participant.

Four patients recommended that information be posted as a step-by-step approach for the patient to follow. Examples included: “how to prepare for appointments and what to expect from the procedures”, “How-to videos” and “Videos that explain process, concerns that individuals may have” and the use of “interactive tools”.

Information on emotional support was suggested to be included online by 3 participants who were hoping for more information on “changing direction and moving on”, “emotional support and counseling” and “coping strategies”.

Patient stories were suggested by 3 participants who recommended “real patients speaking about their journey”, including both stories of “failure and success”.

Other suggestions from 4 patients included: “text - discrete reading”, “women during pregnancy”, “studies and regular updates” and one commented on having difficulty accessing the information due to the website not working well.

Three patients answered “not sure”, one of whom suggested needing some more time to look at what information was already posted.

## Discussion

This study evaluated the use of online fertility-related educational material for patients seeking care for the first time at an academic, urban fertility clinic. Ninety-nine percent of respondents who viewed the information found it helpful and the majority felt it helped prepare them for making treatment decisions. During the study period, many other fertility clinic patients inquired about the postcards referring to the online information and asked for a copy so they could also view the information, resulting in a requirement to order more postcards to be printed to be able to complete the study recruitment. This suggests to us that our information is potentially useful to a much wider range of patients at varying points in fertility treatment, and not just new fertility patients.

Participants in the study were particularly in favour of the online videos and tended to access them through the clinic website rather than through social media. Given the positive feedback provided by patients, we have since starting showing these videos in our clinic waiting room. The videos have also become more prominent on our social media accounts where they have received over 5000 views on YouTube. Given how useful online information is for our patients, we planned to expand the repertoire of information online, using some of the suggestions that patients provided in the survey including patient stories, step-by-step instructions on fertility treatment, interactive online tools and statistics on pregnancy rates. Our clinic has more recently developed detailed online educational modules to replace in-person sessions as part of the routine consent process for patients undergoing IVF and pre-implantation genetic testing.

The intention of this research study was to evaluate online resources to better inform our patients so that they could be more involved in treatment decision-making. While we had hoped for more than 42% of surveyed patients to have viewed the educational material provided, this is similar to another study in the literature of bowel preparation before colonoscopy, in which they tracked that only 49% of patients viewed the mandatory video in advance of the procedure [24]. A lack of time was the most common reason that our patients gave for not viewing the online material. Most follow-up appointments were 4 to 8 weeks after the initial consultation, which should have been ample time to view the material. So while patients stated a lack of time as their primary reason for not accessing the information, it is likely that there were additional factors at play, possibly that patients were not consciously aware of such as the stress of fertility treatment, that they did not feel comfortable admitting on a survey, or were not options on the survey. Since the time of this study, we have made the online information more obvious and accessible to patients at various stages in their fertility journey by having the videos available on YouTube and playing them regularly on the television in the patient waiting room. We are also considering sending out a digital version of the information card to patients through email or the patient portal to remind patients look at the online material the week before the scheduled appointment as an additional way to reach out to patients.

Interestingly, 119 respondents answered questions about the online material even though only 106 stated they had looked at it. This is a limitation of a self-reporting survey in which there is an inherent recall bias. Patients may be viewing lots of different information between their first and second visits and may not remember which information was directly from the fertility clinic. It would have been ideal if we had been able to track exactly which patients accessed the materials directly and what material in particular they looked at. Additionally, we only captured new patients with our survey, but many more existing clinic patients viewed the information during the study period. Our study did not capture the usefulness of the online information for other patients who viewed the information during that time and is a significant limitation of the study. Despite this limitation, an overwhelming majority of patients who claimed they had looked at the online fertility information found the information helpful, stated that it better prepared them for making fertility treatment decisions, and wanted more educational material to be posted online. This finding is reassuring that the videos have achieved the goal of providing educational material that promotes better informed patients and therefore patient care. Since the completion of this study, patients have continued to comment on the usefulness of these videos which are still viewed by many of our patients every day.

While we had anticipated a difference in the demographics of people who stated they had looked at the online educational material provided compared to those who did not, we were surprised to find no significant differences in age, gender, educational level, or ethnicity between groups. This is likely due to the fact that we had very few male participants in the study, a highly ethnically diverse patient population, and a highly educated group of participants who are likely very comfortable with accessing online information. This is consistent with studies in other areas of medicine that have found that seeking medical information online has become increasingly more common before surgical procedures and medical imaging procedures [6, 25]. In a large survey study of people accessing information on the National Health Services website in the United Kingdom, those looking for online medical information were more likely to be female, younger and have a university degree or higher qualification which represents the majority of participants in our study [26].

While videos cannot replace the importance of counselling from a physician, our finding that patients favour videos over written hand-outs and verbal instruction is in keeping with other studies [23, 27, 28]. The preference for videos in our study may be a limitation of the study design in which the videos were more prominent on the various sites than the information provided in text form. Despite the limitation of needing to have access to a computer and the internet in order to view videos, there was a strong preference for videos over text which is likely a true reflection of patient preference. Showing the videos in the clinic waiting room has proven to be an excellent way to improve equity of access to this educational material where limited access to computers due to economic disparities exist.

Previous studies assessing physician-created patient educational videos and computer applications have been positive. Educational videos and other online resources have been shown to improve knowledge scores on questionnaires in multiple different areas of internal medicine and dentistry [17–20, 22]. A review of hospital-based video interventions demonstrated that 61% reported a positive effect of educational videos on patient outcomes compared with control groups using 3 types of video styles: animated presentations, professionals in practice, and patient narratives [29]. Some studies have shown that online educational tools can actually improve disease outcomes such as reduced inhaler misuse in asthma patients, improved medication adherence and weight loss in heart failure patients, and higher rates of satisfactory bowel preparation for colonoscopy patients [24, 30–32]. Patient educational videos can also reduce patient anxiety about medical treatments and help patients to feel better prepared for medical treatment such as in prostate cancer radiotherapy [33, 34]. In our study, patients felt better informed when discussing treatment options with their physician after watching the educational videos which is one of the desired outcomes in creating educational material for our patients. Specifically, we hoped that if patients had already established a foundation of fertility education, this would allow for a more personalized discussion with the patient and more time to address specific patient questions and concerns during the patient-physician encounter, instead of using this valuable time on explaining the basic logistics and reasons for different fertility interventions. Patients also commented that they would like to see instructional videos online giving a how-to approach to fertility investigations and treatment, suggesting that patients would like to make the fertility treatment process more clear and easy to follow.

Fewer patients than expected accessed the educational materials via social media. It is already known that patients can benefit from the use of social media in terms of education, networking, the receipt of support, health care goal setting, and the ability to track personal progress [16]. Social media has been documented to improve patient care in orthopedics and diabetes care [35–37]. This lower than expected uptake may be due to a pre-existing familiarity with our clinic website or a concern about privacy when using a social media account. At the time of the study, the fertility clinic had not been posting on social media accounts for a long period of time and this may have limited patients’ familiarity with viewing fertility educational material in this manner. That being said, some patients commented on a desire for patient stories to be posted online, suggesting a desire for information to be more relatable through narrative which could be well achieved through social media. Since this study, our clinic has continued to focus posting patient educational initiatives on the clinic website while also maintaining content on our social media platforms which have become more popular with time.

People with infertility have often received insufficient information on the subject of fertility and infertility and are motivated to learn more [38, 39]. The present study aimed to specifically improve patient knowledge between the initial consultation and the first follow-up appointment, at which time their test results are reviewed and key fertility treatment decisions are made. The timing of an educational intervention is important as the benefits of online educational tools are often limited to short term benefits with knowledge retention waning over time [20, 21, 40]. In a study of patients with infertility who were advised to read online fertility posts, there was a significant increase in knowledge scores immediately after viewing the online posts, but patients’ beliefs and knowledge levels largely returned to pre-intervention levels at six months [41]. This suggests that online interventions may be helpful for immediate decision-making, but not for long-term knowledge retention. In our clinic, we intended to use the online information to improve the quality of subsequent patient-physician interactions. Giving patients foundational knowledge about fertility should allow for a more personalized discussion of treatment options instead of general counselling. Not only did patients in the study state that the videos were helpful, but the authors have found that the quality of clinic visits with patients have greatly improved since this intervention.

Since the completion of this study, many patients arrive for their initial consultation having already watched the videos on the clinic website, social media accounts or from the television screen in the clinic waiting room. As a result, we have been working to create more online educational content and to engage our patients in designing that content based around their needs.

## Conclusions

This study found that patients attending a fertility clinic, regardless of gender, ethnicity or level of education, found the provision of online fertility information posted on our fertility clinic website helpful and better prepared them for making fertility treatment decisions. Patients were in favour of adding more content to the website, focussing on common fertility problems as well as patient stories and instructional videos. The future of health care is digital and patients and physicians expect to be able to access healthcare resources from their computers, tablets and phones wherever they are. The more physicians are able to provide information virtually and through modern methods such as social media, the more likely we are to be able to relate to our patients and provide them with the care they expect in the modern era. Our study shows that patients welcomed an online approach to fertility education and were keen for this to be developed further.

## Supplementary information

**Additional file 1.** Educational Material.

**Additional file 2.** Online Fertility Educational Material Survey.

## Data Availability

The datasets used and analysed during the current study are available from the corresponding author on reasonable request.

## Abbreviation

QR code: Quick Response code
