# Supplementary material for: Fertility patients’ use and perceptions of online fertility educational material
Source: Fertil Res Pract. 2020 Jul 18;6:11. doi: 10.1186/s40738-020-00083-2 (PMC7368747; doi:10.1186/s40738-020-00083-2)

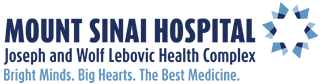


**Optimizing Natural Fertility**

There are many simple things that men and women can do in their daily lives to not only improve fertility, but also general health and well-being of themselves and their future children. Below is a list of some of the most common lifestyle changes that can improve fertility.

**Healthy Weight**

Maintaining a healthy weight is important in improving pregnancy outcomes. Women who are overweight or obese are at higher risk of infertility, miscarriage and lower pregnancy rates with IVF. Overweight and obese women are also at higher risk of pregnancy complications such as high blood pressure, gestational diabetes, Caesarean section, large babies, birth trauma and stillbirth. It is also unsafe for women who are very obese to have sedation which is necessary for IVF, and because of this safety risk, our clinic cannot offer IVF to women with a BMI over 40. The good news is that women who are overweight and not ovulating regularly can improve their chances of pregnancy by losing weight and pregnancy rates. Men who are overweight also have lower fertility and poorer sperm quality compared to men of normal weight.

For men and women wanting to lose weight, fad diets are usually not good for maintaining long-term weight loss. Slow and steady changes in improving diet and increasing exercise are more beneficial. If needed, your doctor can refer you to a dietician or a doctor who specializes in weight management to help you to lose weight in a healthy way.

Both men and women who are underweight can also have difficulty getting pregnant. Women who are underweight are also at risk of delivering prematurely.

For men, a diet rich in carbohydrates, fibre, fruit, vegetables and antioxidants can improve fertility. For women, a diet that is high in iron, low in carbohydrates and low in trans fats is associated with improved fertility. Regular exercise can improve sperm parameters in men and general fertility in overweight women. However, excessive daily exercise in women can be detrimental to the reproductive system. Overall, eating a healthy well-balanced diet and doing regular moderate exercise is important for overall health as well as reproductive health.

**Quitting Bad Habits**

All men and women trying to conceive should quit smoking. Not only is smoking bad for general health, but it also has negative effects on sperm and egg quality. Men and women who smoke have lower chances of achieving pregnancy on their own and with fertility treatment. Smoking in pregnancy also has negative effects including high blood pressure, preeclampsia, hemorrhage and stillbirth. It is also a good idea to quit smoking before having a child since exposure to second-hand smoke can have poor health effects for children including severe asthma. If you need help quitting smoking, see your family doctor and ask about nicotine replacement and other medications to help you quit.


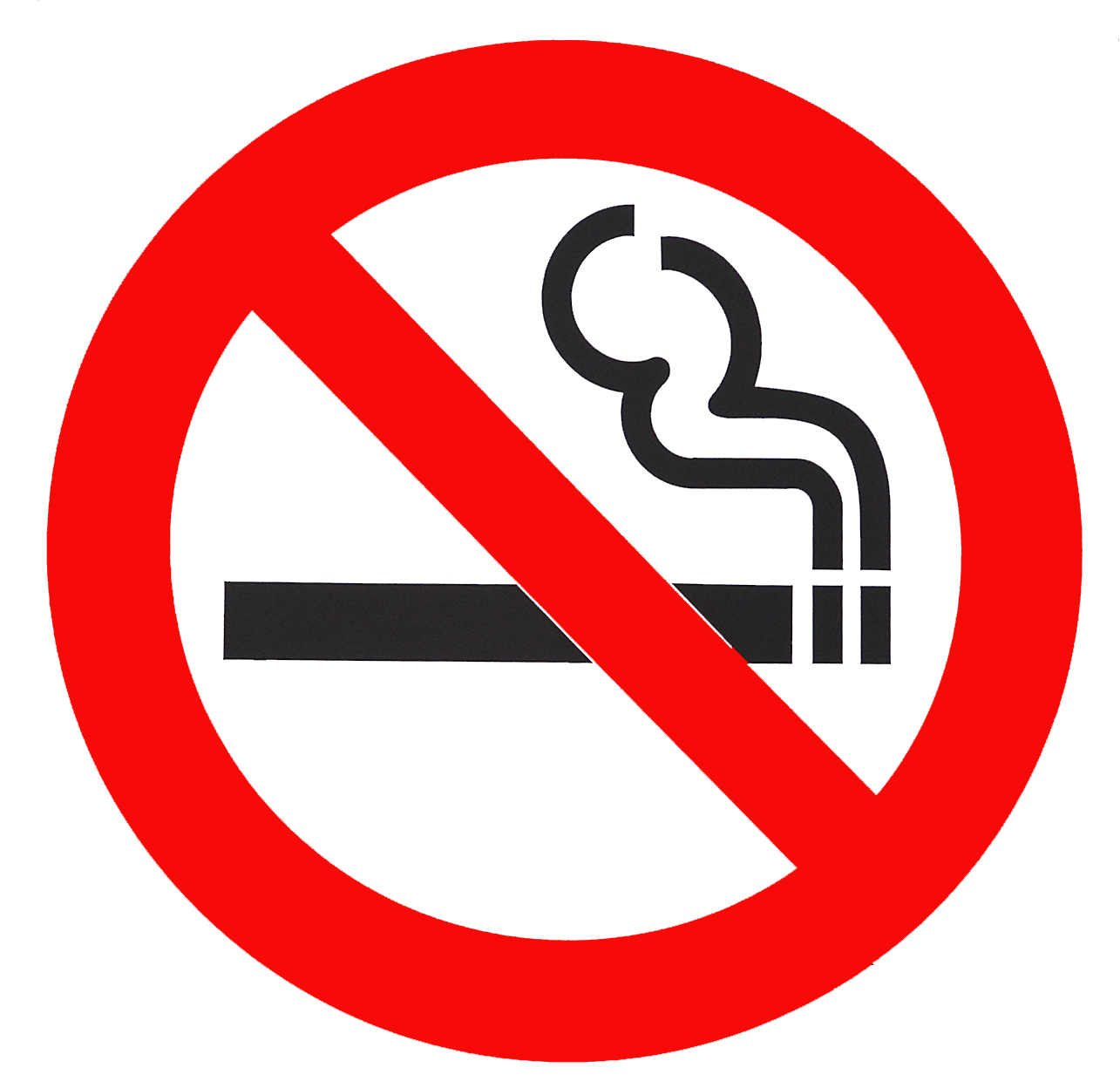


Use of street drugs can have negative consequences for general health, fertility and pregnancy. Even marijuana can cause a significant reduction in testosterone levels and sperm quality which can last up to 3 months, and a reduction in female fertility. While the effect of other drugs on fertility have not been studied, all men and women trying to conceive should quit all illicit drugs.

Alcohol may have some negative effects on fertility for both men and women when used in large amounts. While studies on the safe amount of alcohol consumption are mixed, general good advice is for women to limit themselves to no more than 5 drinks per week and men to no more than 10 drinks per week. There is no known safe level of alcohol consumption in pregnancy so all women should avoid alcohol once they know they are pregnant to avoid the risk of fetal alcohol syndrome (FAS).

Need your morning caffeine intake? While you don’t have to give up your daily cup of brew, some small studies suggest that drinking more than 3 cups of coffee a day can have a negative impact on fertility, miscarriage and stillbirth.

**Vitamins and Supplements**

All women wanting to become pregnant should be taking folic acid 1mg daily, either on its own or as part of a daily prenatal multivitamin. Some women will need to take a higher dose of folic acid 5mg daily – your doctor will advise you if this is necessary. Folic acid is important in reducing the risk of congenital abnormalities such as spina bifida, a spinal cord abnormality.

Your doctor may recommend that you take Coenzyme Q10, a vitamin that may improve the energy in aging eggs. If so, you should take 300mg of soft gel capsules twice a day.

For men with slight abnormalities in the semen analysis, your doctor may recommend that you take a male fertility multivitamin which can be purchased through our clinic. Alternatively, you can take a combination of the following over-the-counter vitamins: vitamin C, vitamin B9, vitamin B12, vitamin E, selenium and zinc.

**Restrictive Clothing and Heat**

High temperature in the scrotum can inhibit the production of sperm. Men should avoid tight fitting underwear, hot tubs, Jacuzzis and saunas.

**Lubricants**

While lubricants can improve pain at intercourse due to vaginal dryness, some can have a detrimental effect on sperm. Lubricants to avoid during the fertile period include olive oil, saliva, Astroglide, FemGlide and Replens. Lubricants that have been shown to not have a negative impact on semen analysis include mineral oil and canola oil.

**Reducing Stress**

Stress can worsen fertility outcomes for both men and women. The diagnosis of infertility and fertility treatment itself can also be very stressful. It can also cause significant strain on relationships. Make sure that you take time for yourself, whether it’s a day at the spa, a night out with friends, exercise, or whatever makes you feel good. It’s also important to take time to do something nice with your partner and remember that you’re in this together.


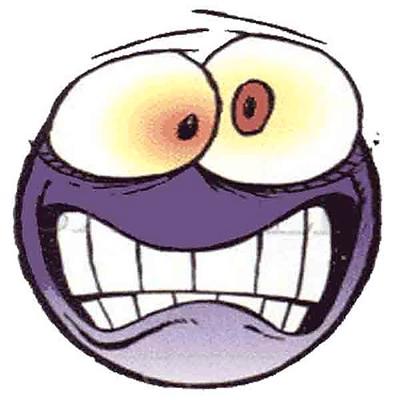


If the stress if becoming overwhelming, please talk to your doctor or nurse so that we can help direct you to the resources that are most likely to help, whether it be social work, psychiatry, sex therapy or accupuncture. Women with anxiety or depression who receive support and counseling can improve their chances of becoming pregnant. We have a social worker on site who is available to all of our patients.

**Timing Intercourse**

The most fertile period is the couple of days before ovulation. Ovulation occurs approximately 14 days before the next period. So, if your periods generally come every 28 days, then it’s best to have intercourse every 1-2 days from days 10 to 14 of your cycle to cover the most fertile period. If cycles are generally every 32 days, you should be having intercourse every 1-2 days from days 14 to 18 to cover the most fertile period. Some women will choose to use ovulation kits or monitors to detect the most fertile period. When the kit or monitor goes positive, the optimal time to have intercourse is that day and the next day.

**References**

Sharma R et al. Lifestyle factors and reproductive health: taking control of your fertility. Reproductive Biology and Endocrinology. 2013: 11;66.


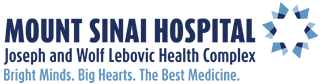


**Infertility Causes and Investigations**


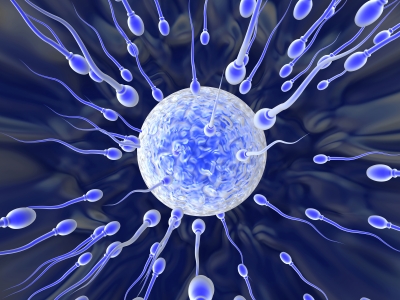


**What do you need to get pregnant?**

- An egg being released each month (ovulation)
- A normal amount of motile normal-shaped sperm
- Open tubes for the egg and sperm to meet for fertilization to occur
- A normal uterus for the embryo (fertilized egg) to implant

**What are causes of infertility in couples seeking fertility treatment?**


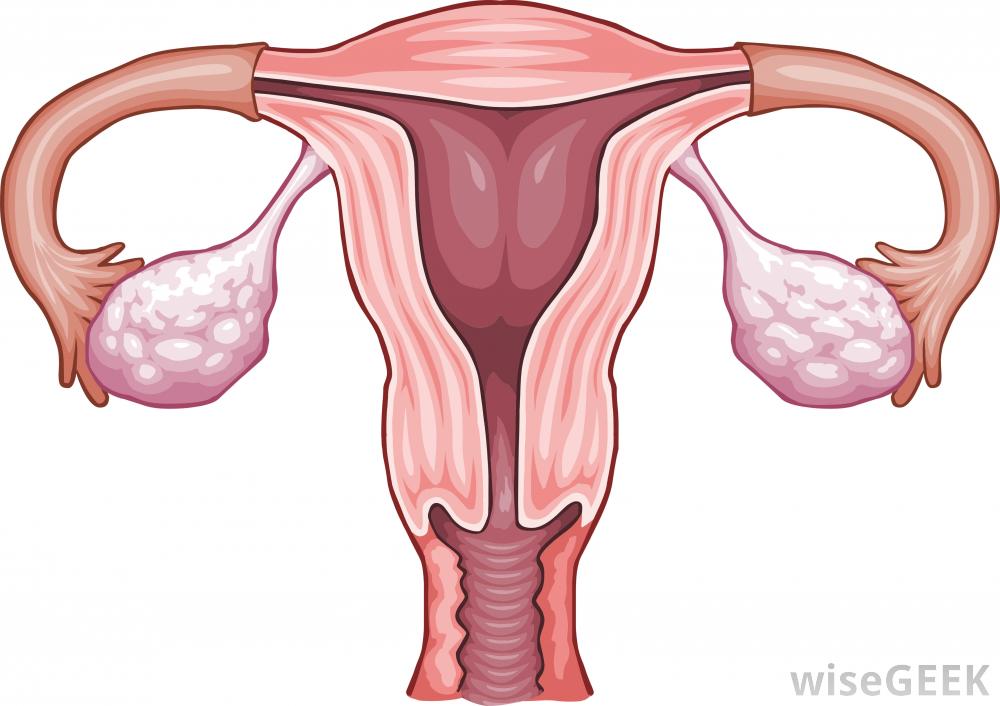


- 35% - male problem
- 35% - fallopian tube problem
- 15% - problem with ovulation
- 10% - unexplained infertility
- 5% - unusual disorder

**What are different tests that your doctor may order?**

- Ovulation tests:
  - A blood test on the 3^rd^ day of your period tests for the resting hormone levels. Some of these include: TSH, Prolactin, FSH, LH, Estradiol, and androgens
  - A progesterone level on day 21 or later of your cycle. It will be high if you ovulated that month
  - Transvaginal ultrasound and blood work monitoring to follow the growth of a follicle, the fluid sack that the egg grows in before being released each month, and the corresponding hormone levels
- Tests for ovarian reserve, an assessment of the number of eggs in the ovary
  - AMH – a blood test done any day of the menstrual cycle where the higher the number, the better the ovarian reserve
  - Day 3 FSH – a blood test on the 3^rd^ day of menstruation tests the response of the ovaries to stimulation from hormones from the brain. If the FSH level is high, this means there is a poor ovarian reserve
  - Antral follicle count – this is measure of the number of resting follicles that we can count by transvaginal ultrasound. The greater the number, the better the ovarian reserve
- Tubal and uterine cavity tests:
  - Hysterosalpingogram (HSG) – this is an x-ray test using dye inserted through the cervix with a metal catheter while pictures are being taken as the dye flows through the uterine cavity and tubes
  - Sonohysterogram – water and air are pushed through a plastic catheter in the uterus while the gynecologist watches with a transvaginal ultrasound probe as the water flows through the uterine cavity and tubes


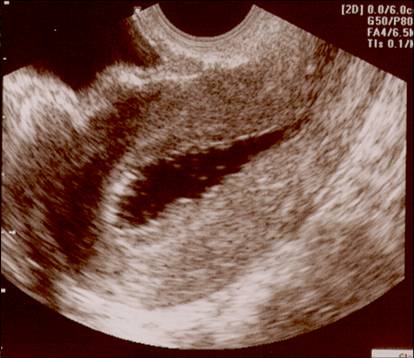

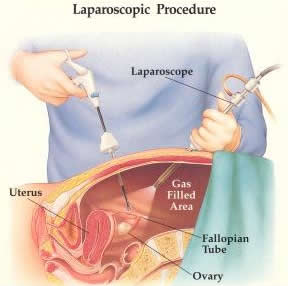


- - Laparoscopy – this is a surgical procedure in which blue dye is pushed through the tubes from the cervix while watching with a camera inserted through the belly button
  - Hysteroscopy – this test uses a camera inserted through the vagina and cervix into the uterus using fluid to see any abnormalites in the uterus such as polyps or fibroids. It does not test the tubes
- Male tests
  - A semen analysis will be ordered for all men. A collection is made into a small container after masturbation and processed in the lab which approximates the number of sperm in the sample, how many are moving well, and how many appear normal. If abnormalities are found, you doctor may order other tests including male hormones, an ultrasound, and genetic tests
- Viral blood work testing
  - All patients must have testing for viral diseases including HIV, hepatitis B and C, and Syphyllis before starting any treatment and these tests must be updated every 12 months

**What are different treatment options for infertility?**

- It depends on the cause:
  - Male factor infertility can be treated with lifestyle changes, surgery, intrauterine inseminations (IUI), in vitro fertilization with intracytoplasmic sperm injection (ICSI), or donor sperm depending on the condition
  - Tubal factor infertility can be treated with surgery or in vitro fertilization (IVF)
  - Ovulatory infertility can be treated with medications such as clomiphene citrate, injectable medications called gonadotropins, or IVF

Unexplained infertility can be treated with controlled ovarian hyperstimulation with timed intrauterine insemination (COH-IUI), IVF or ICSI.


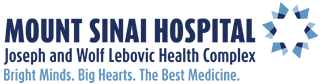


**Polycystic Ovarian Syndrome**


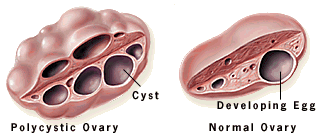


**What is PCOS?**

- A condition in which women have irregular periods, acne, hair growth, and ovaries with lots of little cysts on ultrasound

**How common is it?**

- 5-10% of reproductive age women have PCOS

Most women with the condition have a family history of PCOS, obesity or diabetes

**How is PCOS diagnosed?**

- To meet the diagnostic criteria for PCOS, a women must have at least 2 of the following:
  - Polycystic appearing ovaries on ultrasound
  - No periods or irregular periods
  - High male hormones on blood work or signs of high male hormones on physical exam (acne and hair growth on face, back, chest etc) with no other cause for high male hormones

**Why does PCOS cause infertility?**

- In a normal ovulating woman, each month, the brain sends a signal called FSH to the ovary which stimulates the follicles in the ovary to grow. One follicle will grow bigger than the others and the others will eventually die away. The big follicle will then burst, releasing the egg in a process called ovulation. The egg gets picked up by the tube and if it doesn’t become fertilized by sperm, then a period will follow 2 weeks later.
- In PCOS, there is abnormal signaling between the brain and the ovaries. This is due to high levels of male hormones called androgens. Because the ovaries don’t respond to appropriately to FSH from the brain, there are lots of follicles which will start to grow but get stuck in development and do not grow big enough to ovulate or only do this infrequently. As a result of this disruption of the signaling pathway between the brain and the ovary, women will have no periods or infrequent periods and consequently have difficulty getting pregnant because they are not releasing an egg each month.

**What are some tests that doctors may order to look for PCOS?**

- Tests to rule out other causes of irregular periods such as thyroid, prolactin and rare conditions such congenital adrenal hyperplasia
- Hormone levels
- Cholesterol levels
- Diabetes test

**What are other potential health issues related to PCOS?**

- Obesity
  - Obesity is very common among women with PCOS and worsens the condition
- Gynecologic conditions:
  - Irregular bleeding, heavy bleeding
  - Overgrowth of the lining of the uterus which can be a precursor to uterine cancer
  - Infertility
- Pregnancy- related:
  - Miscarriage
  - Gestational diabetes
  - High blood pressure
- Metabolic:
  - Male-pattern hair growth
  - Hair loss
  - Acne
- Other health considerations:
  - Type 2 diabetes
  - Heart disease

**What are treatment options for managing hair growth in PCOS?**

- Shaving, waxing, electrolysis, laser, Vaniqua
- Birth control pill
- Antiandrogens such as spironolactone, cyproterone acetate, flutamide, and finasteride

**How do we treat irregular periods?**

- If a woman does not want to be pregnant, then it is important to prevent uterine cancer by either using a birth control pill, a Mirena intrauterine system, or a progestin such as Provera or Prometrium on a regular basis. These medications are used for women who are not trying to get pregnant.

**What are different treatment options for women who want to get pregnant?**

- Weight loss! Losing only 5% of body weight can help restore ovulation
- Clomiphene citrate (Clomid or Serophene) is the most commonly used drug to get women to ovulate regularly
- Letrozole (Femara) has been successfully used to bring on ovulation but it is not currently approved for this use in Canada
- Metformin can be used alone or in combination with clomiphene citrate
- Injectable medications (Gonal-F, Puregon, Bravelle) to induce ovulation
- Surgery with laparoscopic ovarian drilling to bring on ovulation
- In vitro fertilization (IVF)

**What are important things to consider with a diagnosis of PCOS?**

- Stay healthy – lose weight, exercise and eat a healthy well-balanced diet to help prevent infertility, pregnancy complications, diabetes, high cholesterol and heart disease
- See your family physician regularly to check for potential long-term complications


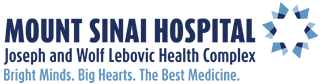


**Common Fertility Drugs**

**Clomiphene Citrate (*Clomid, Serophene*)**

**How is it used?**

- Clomiphene citrate tricks the brain into producing more follicle stimulating hormone (FSH)
- FSH is a signal to the ovaries to make follicles (fluid filled cysts with an egg inside)
- This drug is a pill that is taken for 5 days, starting day 3 or 5 of the menstrual cycle
- This drug can be used to make women ovulate who don’t normally ovulate
- This drug can also be used to make women who ovulate regularly release more than one egg at the same time, known as controlled ovarian hyperstimulation (COH)


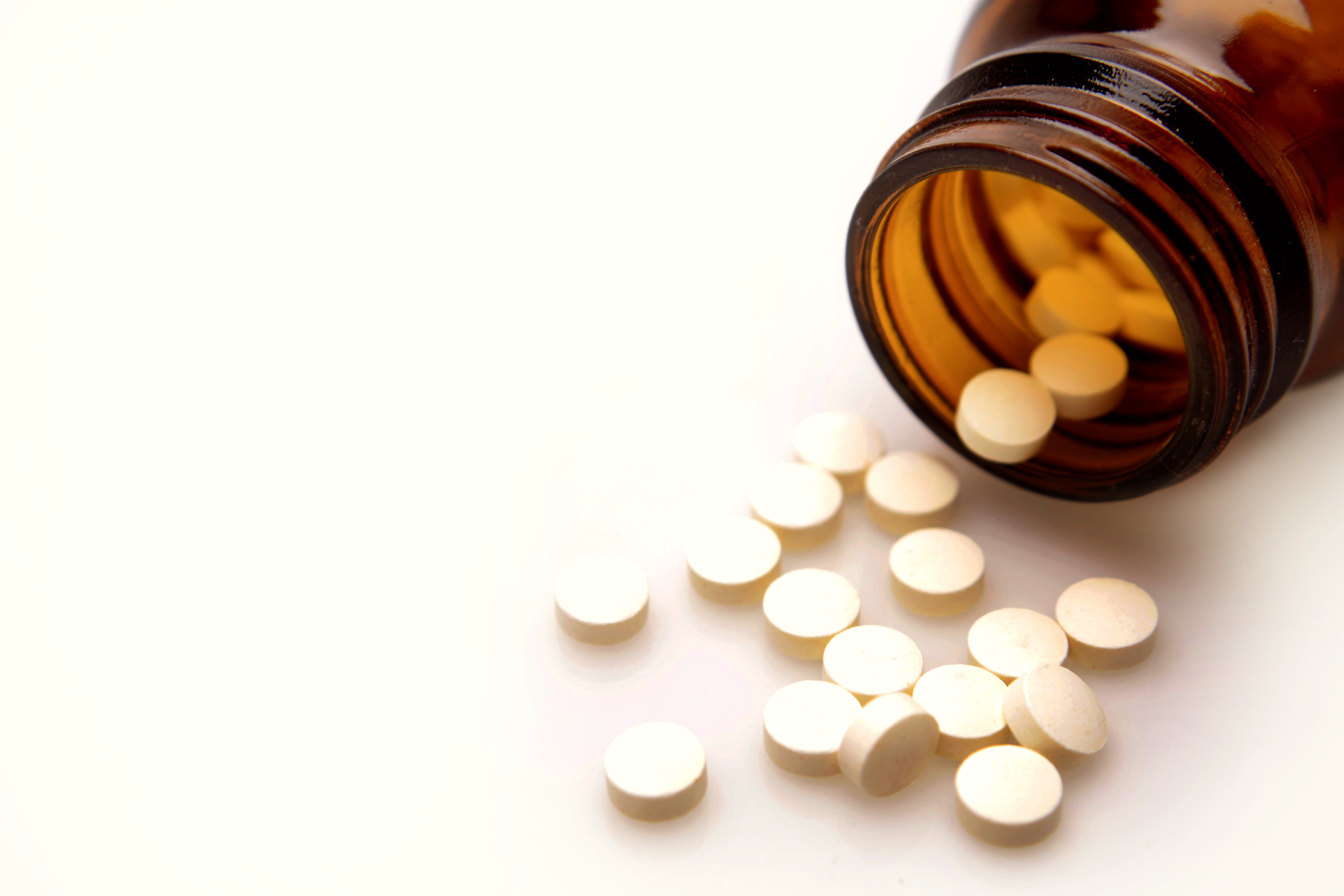


**What are side effects of clomiphene citrate?**

- Ovarian enlargement
- Hot flashes
- Abdominal bloating or discomfort
- Nausea and vomiting
- Breast discomfort
- Visual symptoms
- Headache
- Abnormal uterine bleeding
- Multiple pregnancy (eg twins, triplets or more)

**Gonadotropins (*Puregon, Gonal-F, Bravelle, Menopur, Luveris, Repronex*)**

**How are they used**?

- Gonadotropins refer to the hormones from the brain that stimulate the ovaries named Follicle Stimulating Hormone (FSH) and Luteinizing Hormone (LH)
- These medications are given as daily injections
- These hormones directly stimulate the ovaries to produce follicles with eggs. In small doses, these medications are given to stimulate women who do not ovulate regularly to ovulate in a process called ovulation induction
- In small doses, these medications can be given to produce multiple eggs for controlled ovarian hyperstimulation (COH)
- In larger doses, these medications are given to produce multiple eggs in an IVF cycle

**What are side effects of gonadotropins?**

- Pain, bleeding, redness and bruising at the injection site
- Headache, nausea, vomiting, bloating, abdominal discomfort, breast tenderness, flushing, diarrhea
- Ovarian hyperstimulation syndrome

**GnRH Agonists (*Lupron, Suprefact*) and GnRH Antagonists (*Cetrotide, Ganirelix*)**

**How are they used?**


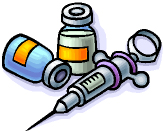


- GnRH agonists and antagonists are used to prevent ovulation during an IVF cycle
- They are most commonly given as daily injectable medications

**What are side effects of GnRH agonists and antagonists?**

- Pain, bleeding, redness and bruising at the injection site
- Headache, nausea

**Ovulation and Egg Maturation Drugs (*HCG, Pregnyl, Ovidrel*)**

**How are they used?**

- These drugs are given to make the ovary release an egg, called ovulation
- These drugs are also given during an IVF cycle to make the eggs undergo the final maturation process, approximately 35 hours before the egg retrieval

**What are side effects of these drugs?**

- Pain, bleeding, redness and bruising at the injection site
- Nausea, vomiting and abdominal pain

**Are fertility medications associated with birth defects?**

- Although this area remains controversial, there is currently no strong evidence that fertility medications are associated with an increased number of birth defects
- Couples who are infertile or require therapy to achieve a pregnancy may have a small increased risk of birth defects that may be unrelated to the use of fertility medications
- The risk of chromosome related abnormalities increases with a woman’s age

**Are fertility medications associated with ovarian cancer?**

- There is conflicting data on the association of fertility medications and ovarian cancer
- Earlier studies initially showed an increased risk of ovarian cancer which has been refuted in more recent data

Infertility itself and lack of pregnancies may be associated


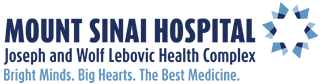


**Assisted Reproductive Technologies**

**Intrauterine Insemination (IUI)**

**What is it?**

- Also known sperm wash, is a procedure in which sperm is processed and then placed into a woman’s uterus through a thin catheter just before ovulation

**What are reasons for doing IUI?**


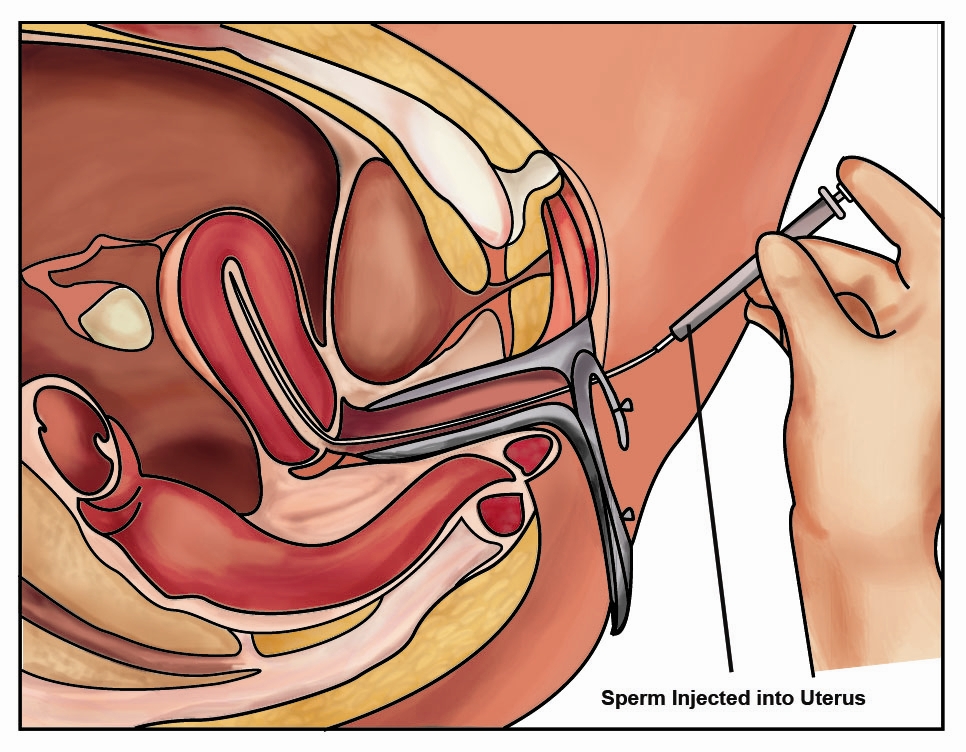


- Male causes of infertility – low sperm count, motility or morphology
- Donor sperm – for male infertility, same sex couples, and single women
- Unexplained infertility

**How does IUI improve pregnancy rates?**

- Timing – by getting concentrated motile sperm in the right place (the uterus) at the right time (just before ovulation)
- Sperm processing - separates and chooses better quality sperm
- Bypassing other issues – cervical mucous problems, lubricants etc

**How do you know when the IUI will be?**

- A woman’s natural or stimulated cycle is monitored by ultrasound and bloodwork or home ovulation sticks until the LH surge is detected or until ovulation is triggered with an injection medication called Ovidrel
- Ovulation occurs on average 36 hours after the start of the LH surge
- The IUI will take place the day after the LH surge or about 36 hours after Ovidrel is given
- If a fresh sperm sample is being used, men will have to provide a sample on the morning of the planned IUI (you will have 1-2 days notice)

**What are the risks of IUI?**

- Some discomfort with the procedure is normal, similar to having a pap test performed
- There is a very small risk of infection. We screen all our patients for sexually transmitted infections to reduce this risk

**In Vitro Fertilization (IVF)**

**What is it?**

- A woman is given injectable medications called gonadotropins in high doses to stimulate the ovaries to make multiple eggs (instead of the usual 1 egg each month)
- Another medication, called a GnRH agonist or GnRH antagonist is given to prevent early ovulation, or the release of the eggs
- Growth of the follicles (fluid filled cysts with an egg inside) is monitored by regular blood work and transvaginal ultrasounds
- When the follicles are big enough to have a mature egg with good estrogen levels, another last injection medication is given to trigger the eggs to become mature
- 36 hours later, the eggs are taken out of the body using a needle attached to a transvaginal ultrasound probe. Women are given pain and sedating medication through an intravenous line to make them comfortable with the procedure
- The same day that the eggs are removed, a fresh sperm sample needs to be provided or a previously frozen sperm sample can be used
- The egg and sperm are combined in the lab using either traditional IVF or ICSI (see below) and growth of the embryo (fertilized egg) is monitored
- Embryos are transferred back into the uterus 3 or 5 days after the egg retrieval. This is done using an abdominal ultrasound to watch as embryos are pushed through a thin catheter into the uterus. No medication is given for this procedure since it is similar to having a pap test done with a full bladder


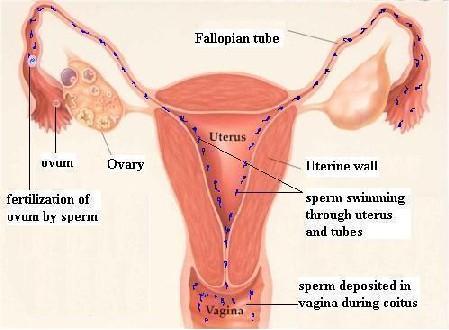

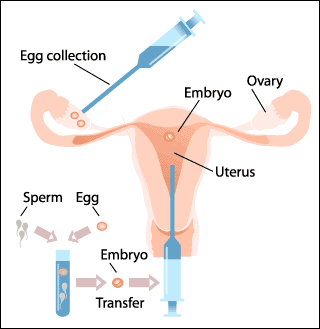


Normal reproduction In-vitro Fertilization

**What are different reasons for doing IVF?**

- There are many different reasons to do IVF some of which include:
  - Blocked fallopian tubes
  - Unexplained infertility
  - Male infertility
  - Genetic or chromosomal disorders
  - Poor ovarian reserve
  - Older age
  - Polycystic ovarian syndrome
  - Endometriosis
  - Egg donation
  - Embryo donation

**How does IVF improve pregnancy rates?**

- IVF improves pregnancy rates in a number of different ways. In an IVF cycle, a woman produces multiple eggs in one cycle which is similar trying naturally many months in a row. Also, the sperm and egg are combined in the lab, bypassing any issues with the fallopian tubes where fertilization normally takes place in the body. Watching the embryos develop in the lab helps us choose which ones are most likely to survive in the body to improve the pregnancy rate in one cycle

**What are the risks of IVF?**

- Ovarian hyperstimulation syndrome (OHSS) – see below Pain, bleeding and infection are risks of egg retrieval
- Nausea, fatigue and memory loss are symptoms of medication given at egg retrieval
- Multiple pregnancy can occur, especially if more than one embryo is transferred back to the uterus

IVF may be associated with an increased risk in congenital abnormalities, although this risk may be related to infertility alone

**Intracytoplasmic Sperm Injection (ICSI)**

**What is it and what is it used for?**


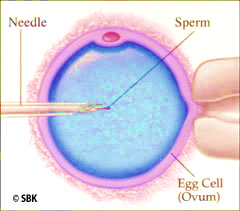


- This is when one sperm is injected into each egg as a form of fertilization after a woman has undergone the stimulation part of an IVF cycle
- Reasons for doing ICSI are male infertility, fertilization problems and preimplantation genetic diagnosis (PGD)
- Some couples will choose to do ICSI for unexplained infertility to avoid the potential for a problem with fertilization at IVF

**What are the risks of ICSI?**

- ICSI has been associated with a slightly higher risk of congenital anomalies in the baby. The risk is increased to about 7-10% compared with 3-5% in the general population

**Assisted Hatching**

**What is it and what is it used for?**

- A laser is used to make a small whole in the shell of the embryo to help it hatch out to improve the chance of implantation in the uterus
- Assisted hatching is performed on all frozen embryos and for embryos with a thick zona pellucida (the shell) which is commonly seen in older women

**Ovarian Hyperstimulation Syndrome**

**What is it and how is it treated?**

- a condition in which the ovaries are over-stimulated, producing leaky blood vessels resulting in symptoms or abdominal bloating and pain, nausea and vomiting, chest pain or shortness of breath, ankle swelling, headache, thirst and voiding less frequently
- Some women will require a procedure to remove fluid from the abdomen which is done with transvaginal ultrasound in the clinic
- In extreme cases, women may need to be admitted to hospital to be given intravenous fluids, medications to prevent blood clots, and procedures to remove fluid which can accumulate in the abdomen and lungs
- Rarely, a woman may need surgery for an ovary that has twisted, called torsion

**How common is it?**

- Mild OHSS is common in 20-33% of IVF cycles
- Severe OHSS occurs in only 0.1-2% of IVF patients

**Preimplantation Genetic Diagnosis and Screening (PGD/PGS)**

**What is it?**

- Samples of cells are taken from embryos created from an IVF-ICSI cycle and are analyzed for genetic disorders such as chromosomal or gene abnormalities
- In the case of a known genetic disorder in the family, patients have the opportunity to only put embryos back into the uterus that do not have the gene mutation to reduce the risk of having a child affected by the genetic disease
- In the case of chromosomal abnormalities, patients have the choice of only putting embryos with a normal balance of chromosomes back into the uterus to reduce the chance of miscarriage or having a child with abnormal chromosomes

**What are reasons for doing PGD/PGS?**

- Men and/or women who are known to be carriers of a single gene disorder such as cystic fibrosis, muscular dystrophy, thalassemia, BRCA
- Men or women who are carriers of a chromosomal abnormality such as a balanced translocation
- Patients who have had multiple IVF cycles with the transfer of multiple blastocysts without achieving a pregnancy
- Women with a history of multiple miscarriages of unknown cause
- Older women who may have an increased risk of having chromosomally abnormal eggs and embryos


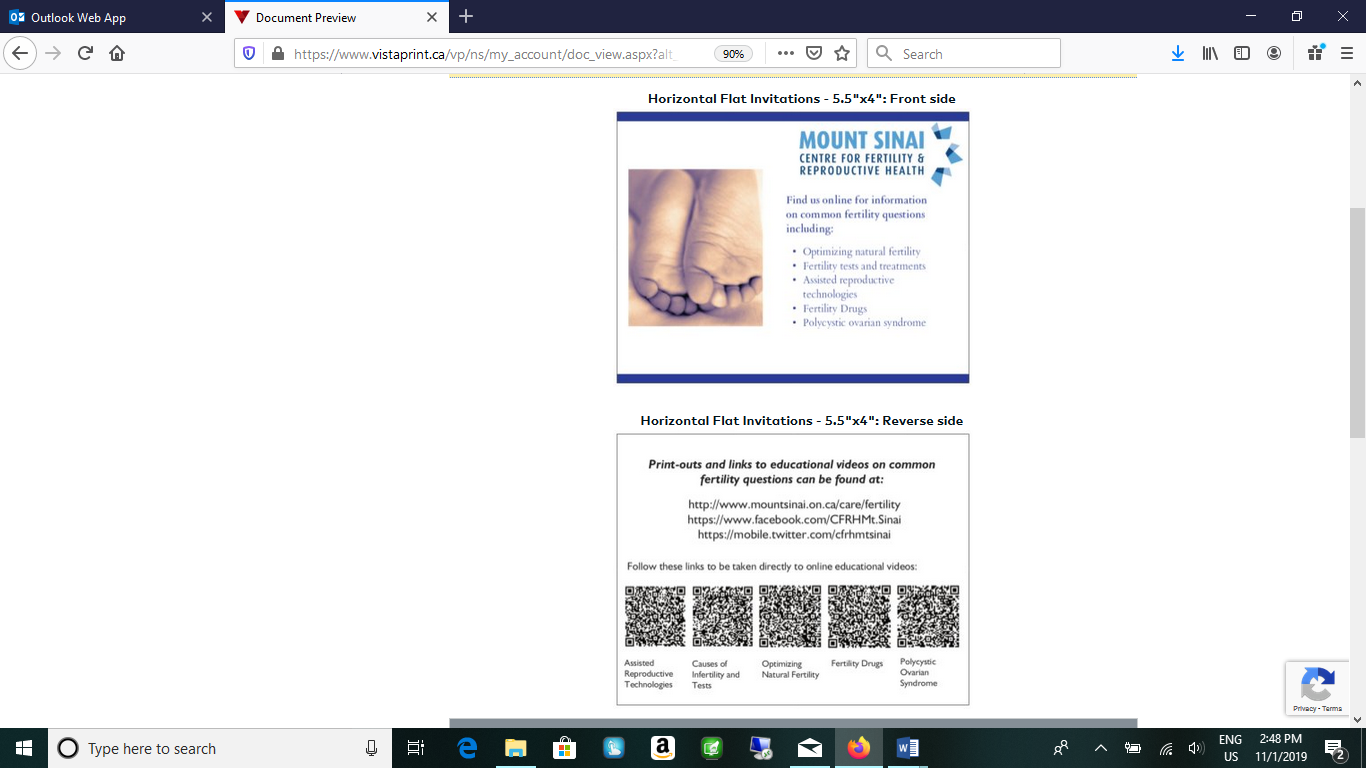

Supplement: Supplementary file 1 — Additional file 1. Educational Material. [file 40738_2020_83_MOESM1_ESM.docx]
