# Supplementary material for: Fertility patients’ use and perceptions of online fertility educational material
Source: Fertil Res Pract. 2020 Jul 18;6:11. doi: 10.1186/s40738-020-00083-2 (PMC7368747; doi:10.1186/s40738-020-00083-2)
Supplement: Supplementary file 2 — Additional file 2. Online Fertility Educational Material Survey. [file 40738_2020_83_MOESM2_ESM.docx]

**Additional file 2**

**Online Fertility Educational Material Survey**

1) What is your age?

__________________________________________________________________________

1. What is your gender?

Male Female

3) What is your ethnic background?

___________________________________________________________________________

4) What is your level of education?

High school College Undergraduate degree Graduate degree Professional degree

5) Were you provided the links to the CFRH clinic website, Facebook and Twitter accounts?

Yes No

6) Did you look at the educational material provided by CFRH online?

Yes No Not yet, but I intend to

*If you answered yes, please go to question 8*

*If you answered no or not yet, please answer question 7, then skip to question 14*

7) Why did you not look at the educational material online?

Not enough time Already well-informed Went to other sites Not interested

8) Where did you go to read the material?

CFRH website CFRH Twitter account CFRH Facebook account

9) Did you find the information helpful?

Very helpful Somewhat helpful Not helpful

10) Was the length of the information appropriate?

Too long Perfect length Not long enough

11) Was the information easy to read?

Very easy to read Easy to read Somewhat confusing Very confusing

12) Which educational material did you look at (please circle all that apply)?

Text Videos

13) Did reading material online before this appointment better prepare you for making fertility decisions today?

Yes No Unsure

14) Which format do you prefer for online educational material?

Text Videos

15) Would you like us to post more educational information online?

Yes No Unsure

Please provide any suggestions of topics that you’d like to see online?

______________________________________________________________________________
